# Bis[dicyclo­hexyl­(phenyl)­phosphane-κ*P*]silver(I) perchlorate dichloro­methane monosolvate

**DOI:** 10.1107/S1600536811009822

**Published:** 2011-03-26

**Authors:** Apollinaire Munyaneza, Reinout Meijboom, Bernard Omondi

**Affiliations:** a Research Centre for Synthesis and Catalysis, Department of Chemistry, University of Johannesburg, PO Box 524 Auckland Park, Johannesburg 2006, South Africa

## Abstract

In the title compound, [Ag{P(C_6_H_11_)_2_(C_6_H_5_)}_2_]ClO_4_·CH_2_Cl_2_, the Ag^I^ atom in the mononuclear complex cation is coordinated by two P atoms of the phosphane ligands [Ag—P = 2.3993 (4) and 2.4011 (4) Å; P—Ag—P = 177.473 (18)°] and the perchlorate anion acts as the counter-anion. There is an Ag⋯O_perchlorate_ inter­action of 2.873 (2) Å, which contributes to the slightly non-linear bond angle about the Ag^I^ atom. Weak inter­molecular C—H⋯O hydrogen-bonding inter­actions involving phenyl, cyclo­hexyl and dichloro­methane H-atom donors and perchlorate O-atom acceptors contribute to the stabilization of the crystal structure.

## Related literature

For a review of the chemistry of silver(I) complexes, see: Meijboom *et al.* (2009 ▶). For the coordination chemistry of Ag*X* salts (*X* = F^−^, Cl^−^, Br^−^, I^−^, BF_4_^−^, PF_6_^−^, NO_3_^−^) with group 15 donor ligands, with the main focus on tertiary phosphanes and in their context as potential anti­tumor agents, see: Berners-Price *et al.* (1998 ▶); Liu *et al.* (2008 ▶). For two- and three-coordinate Ag*X* (*X* = NO_3_^−^) complexes/salts with bulky phosphane ligands, see: Bowmaker *et al.* (1996 ▶); Camalli & Caruso (1988 ▶); Fenske *et al.* (2007 ▶); for *X* = NO_2_, see: Cingolani *et al.* (2002 ▶); for *X* = Cl^−^, Br^−^, I^−^, CN^−^, SCN^−^ and NCO^−^, see: Bowmaker *et al.* (1996 ▶); Bayler *et al.* (1996 ▶); for two-coord­inate *X* = ClO_4_^−^, see: Alyea *et al.* (1982 ▶, 2002 ▶); Baiada *et al.* (1990 ▶); Burgoyne *et al.* (2010 ▶). For the solution behavior of [*L*_n_Ag*X*] complexes, see: Muetterties & Alegranti (1972 ▶). For atomic radii, see: Pauling (1960 ▶).
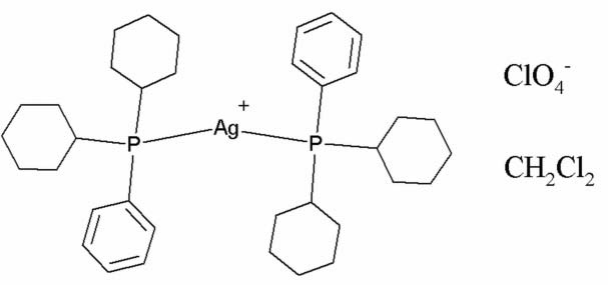


## Experimental

### 

#### Crystal data

[Ag(C_18_H_27_P)_2_]ClO_4_·CH_2_Cl_2_
*M* *_r_* = 840.98
Monoclinic, 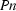

*a* = 9.5910 (3) Å
*b* = 13.4369 (4) Å
*c* = 15.1290 (5) Å
β = 94.706 (1)°
*V* = 1943.15 (11) Å^3^
*Z* = 2
Mo *K*α radiation
μ = 0.84 mm^−1^
*T* = 100 K
0.17 × 0.15 × 0.13 mm

#### Data collection

Bruker X8 APEXII 4K Kappa CCD diffractometer
Absorption correction: multi-scan (*SADABS*; Bruker, 2007 ▶) *T*_min_ = 0.870, *T*_max_ = 0.898
40522 measured reflections
8989 independent reflections
8861 reflections with *I* > 2σ(*I*)
*R*_int_ = 0.025

#### Refinement

*R*[*F* ^2^ > 2σ(*F* ^2^)] = 0.017
*wR*(*F* ^2^) = 0.045
*S* = 1.06
8989 reflections
424 parameters
2 restraints
H-atom parameters constrained
Δρ_max_ = 0.43 e Å^−3^
Δρ_min_ = −0.28 e Å^−3^
Absolute structure: Flack (1983 ▶), 4288 Friedel pairs
Flack parameter: 0.029 (10)

### 

Data collection: *APEX2* (Bruker, 2007 ▶); cell refinement: *SAINT-Plus* (Bruker, 2007 ▶); data reduction: *SAINT-Plus* and *XPREP* (Bruker, 2007 ▶); program(s) used to solve structure: *SIR97* (Altomare *et al.*, 1999 ▶); program(s) used to refine structure: *SHELXL97* (Sheldrick, 2008 ▶); molecular graphics: *DIAMOND* (Brandenburg & Putz, 2005 ▶) and *ORTEP-3* (Farrugia, 1997 ▶); software used to prepare material for publication: *WinGX* (Farrugia, 1999 ▶).

## Supplementary Material

Crystal structure: contains datablocks global, I. DOI: 10.1107/S1600536811009822/zs2098sup1.cif

Structure factors: contains datablocks I. DOI: 10.1107/S1600536811009822/zs2098Isup2.hkl

Additional supplementary materials:  crystallographic information; 3D view; checkCIF report

## Figures and Tables

**Table 1:** Hydrogen-bond geometry (Å, °)

*D*—H⋯*A*	*D*—H	H⋯*A*	*D*⋯*A*	*D*—H⋯*A*
C23—H23*A*⋯O3^i^	0.99	2.48	3.394 (2)	153
C67—H67*A*⋯O2	0.99	2.52	3.423 (3)	152
C13—H13⋯O3^ii^	0.95	2.54	3.448 (2)	160

Symmetry codes: (i) 
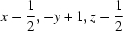
; (ii) 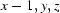.

**Fig. 1.**
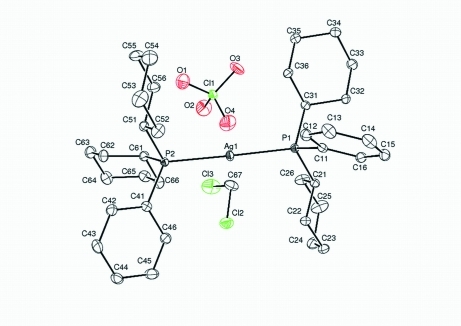
A view of (I) (50% probability displacement ellipsoids) showing atom numbering scheme. H atoms are omitted.

**Fig. 2.**
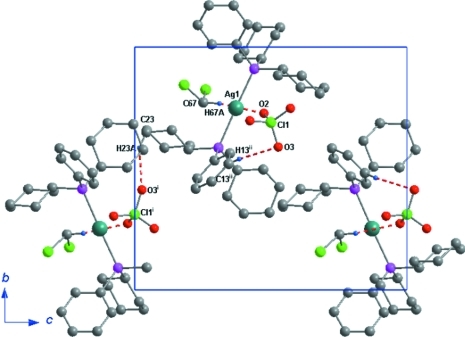
A perspective diagram of the crystal of (I) showing, C—H···O intermolecular interactions.

## supplementary crystallographic information

### Comment

Its been shown that monomeric [AgX(P*R*_3_)_2_]/[Ag(P*R*_3_)_2_]^+^*X*^-^ or dimeric complexes [{AgX(P*R*_3_)_2_}_2_] (Meijboom *et al.*, 2009; Bowmaker *et al.*, 1996 and references therein) are often the products of a reaction of silver(I) salts with monodentate tertiary phosphanes in a 1:2 stoichiometric ratio. The product is dependent on the donor properties of the phosphane ligand, the bulkiness of the ligand substituents and the donor capabilities of the anion and when when π-acid ligands are used in such reactions the complexes formed have been shown to be stable and univalent. These complexes can be two-, three- or four-coordinate depending upon the size and ligation capabilities of the ligands (Baiada *et al.*, 1990). Generally a combination of a weak donor anion and bulky phosphane ligand often leads to the formation of two- or three-coordinate complexes.

In the title compound [Ag{PPh(C_6_H_11_)_2_}_2_]^+^ ClO_4_^-^ . CH_2_Cl_2_ (I) determined at determined at 100 (2) K, the asymmetric unit contains one Ag^I^ complex cation, one perchlorate counter-ion and a dichloromethane molecule of solvation (Fig. 1). In the structure of (I) the cation is mononuclear with the Ag atom coordinated to two P atoms of the dicyclohexylphenylphosphane ligands [Ag—P, 2.3993 (4), 2.4011 (4) Å; P—Ag—P, 177.473 (18)°]. There is an Ag···O_perchlorate_ interaction of 2.8728 (20) Å which contributes to the slightly non-linear bond angle about Ag. This distance indicates very weak electrostatic interaction between the Ag ion and the nitrate counterion (Ag···O distances are 2.873 Å or more). The phosphane ligands appear to also have little steric influnce in the P–Ag–P angle. The cation Ag—P bond distances are well within the Ag—P bond length range for two- or three-coordinate complexes of this type (2.352–2.521 Å). Comparatively, the distances are close to the average of 2.416 (2) Å reported for [Ag{P(C_5_H_9_)Ph_2_}_2_].ClO_4_ (Baiada *et al.*, 1990). Based on the sum of covalent radii of Ag and P atoms, the Ag—P distance is calculated as 2.44 Å (Pauling, 1960).

In the crystal, the Ag complex unit interacts with the perchlorate O atoms resulting in weak intermolecular C—H···O hydrogen-bonding interactions involving phenyl, cyclohexyl and dichloromethane H donors (Table 1), contributing to the stabilization of the structure (Fig. 2).

### Experimental

AgClO_4_ (0.10 g, 0.50 mmol) and P{(C_6_H_11_)_2_Ph} (0.54 g, 1.0 mmol) were dissolved in warm ethanol to give a clear solution which on cooling and solvent evaporation deposited white solid which was then recrystallised in dichloromethane giving colourles crystals of [Ag{PPh(C_6_H_11_)_2_]^+^ClO_4_^-^ in good yield.

### Refinement

All hydrogen atoms were positioned geometrically, with C–H = 0.98 Å for methine H atoms, 0.97 Å for methylene hydrogen and 0.93 Å for aromatic H atoms, and allowed to ride on their parent atoms with *U*_iso_(H) = 1.2*U*_eq_(C).

### Crystal data

**Table:**

[Ag(C_18_H_27_P)_2_]ClO_4_·CH_2_Cl_2_	*F*(000) = 876
*M**_r_* = 840.98	*D*_x_ = 1.437 Mg m^−^^3^
Monoclinic, *P**n*	Mo *K*α radiation, λ = 0.71073 Å
Hall symbol: P -2yac	Cell parameters from 42062 reflections
*a* = 9.5910 (3) Å	θ = 2.4–28°
*b* = 13.4369 (4) Å	µ = 0.84 mm^−^^1^
*c* = 15.1290 (5) Å	*T* = 100 K
β = 94.706 (1)°	Block, colourless
*V* = 1943.15 (11) Å^3^	0.17 × 0.15 × 0.13 mm
*Z* = 2	

### Data collection

**Table:**

Bruker X8 APEXII 4K Kappa CCD diffractometer	8861 reflections with *I* > 2σ(*I*)
graphite	*R*_int_ = 0.025
φ and ω scans	θ_max_ = 28°, θ_min_ = 2.4°
Absorption correction: multi-scan (*SADABS*; Bruker, 2007)	*h* = −12→12
*T*_min_ = 0.870, *T*_max_ = 0.898	*k* = −17→17
40522 measured reflections	*l* = −19→19
8989 independent reflections	

### Refinement

**Table:**

Refinement on *F*^2^	Secondary atom site location: difference Fourier map
Least-squares matrix: full	Hydrogen site location: inferred from neighbouring sites
*R*[*F*^2^ > 2σ(*F*^2^)] = 0.017	H-atom parameters constrained
*wR*(*F*^2^) = 0.045	*w* = 1/[σ^2^(*F*_o_^2^) + (0.0211*P*)^2^ + 0.5077*P*] where *P* = (*F*_o_^2^ + 2*F*_c_^2^)/3
*S* = 1.06	(Δ/σ)_max_ = 0.004
8989 reflections	Δρ_max_ = 0.43 e Å^−^^3^
424 parameters	Δρ_min_ = −0.28 e Å^−^^3^
2 restraints	Absolute structure: Flack (1983), 4288 Friedel pairs
Primary atom site location: structure-invariant direct methods	Flack parameter: 0.029 (10)

### Special details

**Table:**

Experimental. The intensity data were collected on a Bruker X8 Apex 4 K CCD diffractometer using an exposure time of 15 sec/per frame. A total of 2217 frames were collected with a frame width of 0.5° covering up to θ = 28.00° with 99.8% completeness accomplished.
Geometry. All e.s.d.'s (except the e.s.d. in the dihedral angle between two l.s. planes) are estimated using the full covariance matrix. The cell e.s.d.'s are taken into account individually in the estimation of e.s.d.'s in distances, angles and torsion angles; correlations between e.s.d.'s in cell parameters are only used when they are defined by crystal symmetry. An approximate (isotropic) treatment of cell e.s.d.'s is used for estimating e.s.d.'s involving l.s. planes.
Refinement. Refinement of *F*^2^ against ALL reflections. The weighted *R*-factor *wR* and goodness of fit *S* are based on *F*^2^, conventional *R*-factors *R* are based on *F*, with *F* set to zero for negative *F*^2^. The threshold expression of *F*^2^ > σ(*F*^2^) is used only for calculating *R*-factors(gt) *etc*. and is not relevant to the choice of reflections for refinement. *R*-factors based on *F*^2^ are statistically about twice as large as those based on *F*, and *R*- factors based on ALL data will be even larger. >>> The Following ALERTS were generated <<< Format: alert-number_ALERT_alert-type_alert-level text 111_ALERT_2_B ADDSYM Detects (Pseudo) Centre of Symmetry ···.. 91 PerFi 113_ALERT_2_C ADDSYM Suggests Possible Pseudo/New Space-group. P21/c Author Response: The ADDSYM alert is false. For Z = 2, a center of symmetry is impossible. The structure cannot be solved in P21/c. 220_ALERT_2_C Large Non-Solvent C *U*_eq_(max)/*U*_eq_(min) ··· 2.61 Ratio 244_ALERT_4_C Low 'Solvent' *U*_eq_ as Compared to Neighbours for Cl1 Probably caused by movement of carbon atom. 860_ALERT_3_G Note: Number of Least-Squares Restraints ······. 2 Author response: Two reflections omitted (0 1 1) and (0 1 -1); Affected by beam stop.

### Fractional atomic coordinates and isotropic or equivalent isotropic displacement parameters (Å^2^)

**Table:**

	*x*	*y*	*z*	*U*_iso_*/*U*_eq_	
C11	−0.02707 (17)	0.53292 (12)	0.29399 (11)	0.0125 (3)	
C12	−0.12656 (18)	0.56247 (12)	0.35064 (11)	0.0161 (3)	
H12	−0.1041	0.613	0.3933	0.019*	
C13	−0.25890 (19)	0.51870 (13)	0.34549 (13)	0.0204 (4)	
H13	−0.3257	0.5388	0.385	0.024*	
C14	−0.29256 (18)	0.44581 (13)	0.28264 (13)	0.0205 (4)	
H14	−0.3824	0.4155	0.2793	0.025*	
C15	−0.19568 (19)	0.41696 (13)	0.22475 (12)	0.0179 (3)	
H15	−0.2199	0.3681	0.1807	0.021*	
C16	−0.06343 (18)	0.45913 (12)	0.23082 (11)	0.0145 (3)	
H16	0.0033	0.4378	0.1918	0.017*	
C21	0.21578 (17)	0.57881 (12)	0.19859 (10)	0.0119 (3)	
H21	0.1938	0.5116	0.1728	0.014*	
C22	0.14331 (18)	0.65755 (12)	0.13730 (11)	0.0150 (3)	
H22A	0.0407	0.6482	0.1348	0.018*	
H22B	0.1653	0.7248	0.1612	0.018*	
C23	0.19249 (19)	0.64875 (13)	0.04391 (11)	0.0182 (3)	
H23A	0.1622	0.5838	0.018	0.022*	
H23B	0.1485	0.7019	0.0059	0.022*	
C24	0.3509 (2)	0.65726 (14)	0.04524 (11)	0.0246 (4)	
H24A	0.3799	0.644	−0.015	0.03*	
H24B	0.3795	0.726	0.0617	0.03*	
C25	0.4249 (2)	0.58524 (17)	0.11003 (12)	0.0291 (4)	
H25A	0.5268	0.5982	0.1133	0.035*	
H25B	0.4089	0.5165	0.0881	0.035*	
C26	0.37395 (19)	0.59401 (14)	0.20338 (12)	0.0212 (4)	
H26A	0.4209	0.5433	0.2428	0.025*	
H26B	0.3979	0.6605	0.2283	0.025*	
C31	0.24934 (17)	0.49289 (12)	0.37783 (10)	0.0128 (3)	
H31	0.3498	0.5135	0.3804	0.015*	
C32	0.24074 (18)	0.38648 (11)	0.34070 (10)	0.0160 (3)	
H32A	0.1417	0.3649	0.3343	0.019*	
H32B	0.2764	0.3857	0.2811	0.019*	
C33	0.3257 (2)	0.31375 (12)	0.40118 (11)	0.0197 (4)	
H33A	0.312	0.2453	0.3778	0.024*	
H33B	0.4264	0.3302	0.4014	0.024*	
C34	0.28224 (19)	0.31800 (12)	0.49569 (11)	0.0180 (3)	
H34A	0.1849	0.2938	0.4966	0.022*	
H34B	0.3435	0.2737	0.534	0.022*	
C35	0.29225 (19)	0.42345 (12)	0.53194 (11)	0.0153 (3)	
H35A	0.3913	0.445	0.5367	0.018*	
H35B	0.2591	0.4245	0.5922	0.018*	
C36	0.20534 (17)	0.49594 (12)	0.47266 (10)	0.0136 (3)	
H36A	0.2178	0.5642	0.4966	0.016*	
H36B	0.1051	0.4784	0.4725	0.016*	
C41	0.10067 (18)	1.01131 (12)	0.37557 (11)	0.0155 (3)	
C42	0.04090 (18)	1.09320 (12)	0.41545 (11)	0.0180 (3)	
H42	0.0401	1.0958	0.4782	0.022*	
C43	−0.0174 (2)	1.17090 (13)	0.36388 (12)	0.0222 (4)	
H43	−0.0644	1.2236	0.391	0.027*	
C44	−0.00698 (19)	1.17140 (12)	0.27295 (11)	0.0204 (3)	
H44	−0.0393	1.2272	0.2385	0.024*	
C45	0.0506 (2)	1.09062 (14)	0.23249 (12)	0.0224 (4)	
H45	0.052	1.089	0.1698	0.027*	
C46	0.10678 (18)	1.01141 (13)	0.28362 (11)	0.0165 (3)	
H46	0.1494	0.9573	0.2557	0.02*	
C51	0.09940 (17)	0.90840 (12)	0.54482 (10)	0.0134 (3)	
H51	0.1079	0.9762	0.5719	0.016*	
C52	−0.05629 (18)	0.88336 (14)	0.52725 (12)	0.0198 (4)	
H52A	−0.102	0.9336	0.4867	0.024*	
H52B	−0.0666	0.8175	0.4981	0.024*	
C53	−0.1279 (2)	0.88174 (16)	0.61383 (12)	0.0274 (4)	
H53A	−0.1255	0.9493	0.6401	0.033*	
H53B	−0.2271	0.8621	0.6014	0.033*	
C54	−0.0550 (2)	0.80857 (15)	0.67974 (13)	0.0311 (4)	
H54A	−0.0995	0.8117	0.7364	0.037*	
H54B	−0.0666	0.7401	0.6562	0.037*	
C55	0.1000 (2)	0.83198 (14)	0.69654 (12)	0.0253 (4)	
H55A	0.1449	0.7813	0.7369	0.03*	
H55B	0.1115	0.8976	0.726	0.03*	
C56	0.1723 (2)	0.83343 (13)	0.60998 (11)	0.0190 (3)	
H56A	0.1688	0.7662	0.5831	0.023*	
H56B	0.2718	0.8522	0.6225	0.023*	
C61	0.36538 (18)	0.95152 (12)	0.46563 (11)	0.0140 (3)	
H61	0.4118	0.8994	0.505	0.017*	
C62	0.38033 (18)	1.04925 (13)	0.51713 (12)	0.0169 (3)	
H62A	0.3276	1.1021	0.4832	0.02*	
H62B	0.339	1.0414	0.5746	0.02*	
C63	0.5324 (2)	1.08093 (14)	0.53414 (13)	0.0215 (4)	
H63A	0.5806	1.0347	0.5775	0.026*	
H63B	0.536	1.1483	0.5608	0.026*	
C64	0.61025 (19)	1.08221 (13)	0.45012 (13)	0.0227 (4)	
H64A	0.5715	1.1354	0.41	0.027*	
H64B	0.7104	1.0969	0.4658	0.027*	
C65	0.59659 (19)	0.98287 (13)	0.40285 (12)	0.0199 (4)	
H65A	0.6444	0.931	0.4406	0.024*	
H65B	0.6433	0.9866	0.347	0.024*	
C66	0.44409 (18)	0.95370 (13)	0.38199 (11)	0.0178 (3)	
H66A	0.4394	0.8871	0.3538	0.021*	
H66B	0.3986	1.0019	0.3394	0.021*	
C67	0.6908 (2)	0.77168 (16)	0.25252 (15)	0.0267 (4)	
H67A	0.6557	0.7674	0.3122	0.032*	
H67B	0.6956	0.7033	0.2285	0.032*	
O1	0.5106 (2)	0.74765 (10)	0.57255 (11)	0.0428 (4)	
O2	0.68626 (18)	0.72677 (14)	0.47496 (12)	0.0383 (4)	
O3	0.57037 (17)	0.58815 (9)	0.52764 (9)	0.0344 (3)	
O4	0.45640 (16)	0.69645 (12)	0.42368 (10)	0.0393 (4)	
P1	0.14874 (4)	0.58485 (3)	0.30855 (3)	0.01062 (8)	
P2	0.18357 (4)	0.90993 (3)	0.43989 (3)	0.01080 (8)	
Cl1	0.55468 (4)	0.68973 (3)	0.50066 (2)	0.01629 (7)	
Cl2	0.57383 (5)	0.84360 (3)	0.18233 (3)	0.02784 (9)	
Cl3	0.85874 (6)	0.82457 (5)	0.26125 (4)	0.04347 (13)	
Ag1	0.170525 (17)	0.748243 (8)	0.372437 (13)	0.01296 (3)	

### Atomic displacement parameters (Å^2^)

**Table:**

	*U*^11^	*U*^22^	*U*^33^	*U*^12^	*U*^13^	*U*^23^
C11	0.0124 (7)	0.0107 (7)	0.0141 (7)	−0.0004 (6)	0.0002 (6)	0.0027 (5)
C12	0.0167 (8)	0.0149 (7)	0.0170 (8)	0.0018 (6)	0.0023 (6)	−0.0001 (6)
C13	0.0155 (9)	0.0180 (9)	0.0286 (10)	0.0021 (7)	0.0075 (7)	0.0023 (7)
C14	0.0125 (8)	0.0176 (8)	0.0310 (9)	0.0002 (6)	0.0001 (7)	0.0054 (7)
C15	0.0190 (9)	0.0147 (8)	0.0191 (8)	−0.0023 (6)	−0.0039 (7)	0.0007 (6)
C16	0.0160 (8)	0.0145 (7)	0.0130 (7)	0.0014 (6)	0.0012 (6)	0.0002 (6)
C21	0.0117 (7)	0.0122 (7)	0.0120 (7)	−0.0001 (6)	0.0022 (6)	0.0008 (5)
C22	0.0183 (8)	0.0140 (7)	0.0124 (7)	0.0010 (6)	−0.0007 (6)	0.0018 (6)
C23	0.0236 (9)	0.0191 (8)	0.0118 (7)	−0.0031 (7)	0.0003 (6)	0.0019 (6)
C24	0.0275 (10)	0.0308 (10)	0.0162 (8)	−0.0107 (8)	0.0052 (7)	0.0021 (7)
C25	0.0157 (9)	0.0517 (12)	0.0208 (9)	0.0019 (8)	0.0072 (7)	0.0039 (8)
C26	0.0139 (8)	0.0334 (10)	0.0165 (8)	0.0019 (7)	0.0025 (6)	0.0053 (7)
C31	0.0139 (8)	0.0108 (7)	0.0136 (8)	0.0027 (6)	−0.0001 (6)	0.0005 (6)
C32	0.0239 (9)	0.0115 (7)	0.0122 (7)	0.0034 (6)	−0.0011 (6)	−0.0014 (5)
C33	0.0274 (9)	0.0131 (7)	0.0185 (8)	0.0073 (7)	0.0002 (7)	−0.0001 (6)
C34	0.0224 (8)	0.0155 (7)	0.0154 (7)	0.0007 (6)	−0.0027 (6)	0.0023 (6)
C35	0.0169 (8)	0.0156 (7)	0.0131 (7)	−0.0003 (6)	−0.0003 (6)	0.0005 (6)
C36	0.0164 (8)	0.0142 (7)	0.0103 (7)	0.0007 (6)	0.0008 (6)	−0.0007 (5)
C41	0.0181 (8)	0.0136 (8)	0.0148 (8)	0.0013 (6)	0.0005 (6)	0.0009 (6)
C42	0.0209 (9)	0.0172 (8)	0.0156 (8)	0.0037 (6)	0.0000 (6)	−0.0017 (6)
C43	0.0267 (9)	0.0140 (8)	0.0256 (9)	0.0054 (7)	0.0009 (7)	−0.0027 (7)
C44	0.0216 (9)	0.0154 (7)	0.0228 (9)	0.0019 (6)	−0.0067 (7)	0.0024 (6)
C45	0.0267 (10)	0.0242 (9)	0.0164 (8)	0.0034 (7)	0.0016 (7)	0.0049 (7)
C46	0.0165 (8)	0.0161 (7)	0.0167 (8)	0.0026 (6)	0.0005 (6)	−0.0006 (6)
C51	0.0157 (8)	0.0129 (7)	0.0119 (7)	−0.0014 (6)	0.0024 (6)	−0.0011 (5)
C52	0.0127 (8)	0.0264 (9)	0.0207 (8)	−0.0041 (7)	0.0033 (6)	−0.0024 (7)
C53	0.0203 (9)	0.0368 (11)	0.0264 (10)	−0.0034 (8)	0.0098 (8)	−0.0038 (8)
C54	0.0395 (12)	0.0307 (10)	0.0255 (9)	−0.0087 (9)	0.0185 (9)	0.0006 (8)
C55	0.0365 (11)	0.0260 (9)	0.0142 (8)	0.0033 (8)	0.0065 (7)	0.0048 (7)
C56	0.0209 (9)	0.0206 (8)	0.0160 (8)	0.0044 (7)	0.0041 (6)	0.0027 (6)
C61	0.0144 (8)	0.0141 (7)	0.0135 (7)	−0.0027 (6)	0.0010 (6)	−0.0003 (6)
C62	0.0150 (8)	0.0161 (8)	0.0196 (8)	−0.0012 (6)	0.0016 (6)	−0.0032 (6)
C63	0.0229 (9)	0.0198 (9)	0.0214 (8)	−0.0056 (7)	0.0004 (7)	−0.0011 (7)
C64	0.0167 (8)	0.0206 (9)	0.0308 (10)	−0.0038 (7)	0.0016 (7)	−0.0006 (7)
C65	0.0164 (8)	0.0200 (9)	0.0235 (9)	−0.0011 (7)	0.0026 (7)	0.0024 (7)
C66	0.0146 (8)	0.0172 (8)	0.0218 (9)	0.0003 (6)	0.0037 (6)	−0.0029 (6)
C67	0.0241 (11)	0.0246 (8)	0.0309 (10)	−0.0043 (8)	−0.0012 (8)	0.0113 (8)
O1	0.0686 (13)	0.0316 (8)	0.0303 (8)	0.0155 (7)	0.0172 (8)	−0.0063 (6)
O2	0.0294 (9)	0.0453 (8)	0.0407 (9)	−0.0203 (8)	0.0064 (7)	−0.0006 (7)
O3	0.0566 (10)	0.0163 (6)	0.0332 (7)	0.0095 (6)	0.0208 (7)	0.0080 (5)
O4	0.0315 (8)	0.0464 (9)	0.0372 (8)	0.0029 (7)	−0.0137 (6)	−0.0016 (7)
P1	0.01159 (19)	0.00939 (17)	0.01101 (18)	−0.00009 (14)	0.00178 (14)	−0.00049 (14)
P2	0.01190 (19)	0.00915 (18)	0.01123 (18)	0.00047 (15)	0.00028 (15)	−0.00057 (14)
Cl1	0.01967 (19)	0.01178 (15)	0.01761 (17)	0.00017 (14)	0.00265 (14)	−0.00074 (13)
Cl2	0.0276 (2)	0.0304 (2)	0.0245 (2)	−0.00379 (18)	−0.00447 (17)	0.00539 (17)
Cl3	0.0210 (2)	0.0579 (3)	0.0507 (3)	−0.0038 (2)	−0.0021 (2)	0.0135 (3)
Ag1	0.01589 (5)	0.00932 (4)	0.01378 (5)	−0.00066 (4)	0.00196 (3)	−0.00173 (3)

### Geometric parameters (Å, °)

**Table:**

C11—C12	1.392 (2)	C43—H43	0.95
C11—C16	1.401 (2)	C44—C45	1.383 (3)
C11—P1	1.8215 (17)	C44—H44	0.95
C12—C13	1.395 (3)	C45—C46	1.398 (2)
C12—H12	0.95	C45—H45	0.95
C13—C14	1.385 (3)	C46—H46	0.95
C13—H13	0.95	C51—C52	1.532 (2)
C14—C15	1.384 (3)	C51—C56	1.537 (2)
C14—H14	0.95	C51—P2	1.8391 (16)
C15—C16	1.385 (2)	C51—H51	1
C15—H15	0.95	C52—C53	1.528 (2)
C16—H16	0.95	C52—H52A	0.99
C21—C26	1.526 (2)	C52—H52B	0.99
C21—C22	1.535 (2)	C53—C54	1.528 (3)
C21—P1	1.8338 (16)	C53—H53A	0.99
C21—H21	1	C53—H53B	0.99
C22—C23	1.530 (2)	C54—C55	1.520 (3)
C22—H22A	0.99	C54—H54A	0.99
C22—H22B	0.99	C54—H54B	0.99
C23—C24	1.523 (3)	C55—C56	1.532 (2)
C23—H23A	0.99	C55—H55A	0.99
C23—H23B	0.99	C55—H55B	0.99
C24—C25	1.512 (3)	C56—H56A	0.99
C24—H24A	0.99	C56—H56B	0.99
C24—H24B	0.99	C61—C66	1.526 (2)
C25—C26	1.536 (2)	C61—C62	1.528 (2)
C25—H25A	0.99	C61—P2	1.8420 (17)
C25—H25B	0.99	C61—H61	1
C26—H26A	0.99	C62—C63	1.521 (3)
C26—H26B	0.99	C62—H62A	0.99
C31—C36	1.529 (2)	C62—H62B	0.99
C31—C32	1.536 (2)	C63—C64	1.526 (3)
C31—P1	1.8412 (16)	C63—H63A	0.99
C31—H31	1	C63—H63B	0.99
C32—C33	1.528 (2)	C64—C65	1.515 (3)
C32—H32A	0.99	C64—H64A	0.99
C32—H32B	0.99	C64—H64B	0.99
C33—C34	1.523 (2)	C65—C66	1.522 (2)
C33—H33A	0.99	C65—H65A	0.99
C33—H33B	0.99	C65—H65B	0.99
C34—C35	1.519 (2)	C66—H66A	0.99
C34—H34A	0.99	C66—H66B	0.99
C34—H34B	0.99	C67—Cl3	1.756 (2)
C35—C36	1.525 (2)	C67—Cl2	1.768 (2)
C35—H35A	0.99	C67—H67A	0.99
C35—H35B	0.99	C67—H67B	0.99
C36—H36A	0.99	O1—Cl1	1.4291 (15)
C36—H36B	0.99	O2—Cl1	1.4394 (16)
C41—C46	1.397 (2)	O3—Cl1	1.4290 (12)
C41—C42	1.400 (2)	O4—Cl1	1.4397 (15)
C41—P2	1.8187 (17)	P1—Ag1	2.4011 (4)
C42—C43	1.393 (2)	P2—Ag1	2.3993 (4)
C42—H42	0.95	Cl1—O2	1.4394 (16)
C43—C44	1.388 (3)		
			
C12—C11—C16	118.49 (15)	C46—C45—H45	119.9
C12—C11—P1	119.40 (13)	C41—C46—C45	120.41 (16)
C16—C11—P1	121.98 (13)	C41—C46—H46	119.8
C11—C12—C13	120.79 (16)	C45—C46—H46	119.8
C11—C12—H12	119.6	C52—C51—C56	110.67 (14)
C13—C12—H12	119.6	C52—C51—P2	110.07 (11)
C14—C13—C12	119.75 (16)	C56—C51—P2	110.61 (11)
C14—C13—H13	120.1	C52—C51—H51	108.5
C12—C13—H13	120.1	C56—C51—H51	108.5
C15—C14—C13	120.13 (16)	P2—C51—H51	108.5
C15—C14—H14	119.9	C53—C52—C51	110.76 (15)
C13—C14—H14	119.9	C53—C52—H52A	109.5
C14—C15—C16	120.15 (16)	C51—C52—H52A	109.5
C14—C15—H15	119.9	C53—C52—H52B	109.5
C16—C15—H15	119.9	C51—C52—H52B	109.5
C15—C16—C11	120.65 (15)	H52A—C52—H52B	108.1
C15—C16—H16	119.7	C54—C53—C52	110.79 (16)
C11—C16—H16	119.7	C54—C53—H53A	109.5
C26—C21—C22	109.59 (13)	C52—C53—H53A	109.5
C26—C21—P1	111.85 (11)	C54—C53—H53B	109.5
C22—C21—P1	110.02 (11)	C52—C53—H53B	109.5
C26—C21—H21	108.4	H53A—C53—H53B	108.1
C22—C21—H21	108.4	C55—C54—C53	111.37 (15)
P1—C21—H21	108.4	C55—C54—H54A	109.4
C23—C22—C21	110.23 (14)	C53—C54—H54A	109.4
C23—C22—H22A	109.6	C55—C54—H54B	109.4
C21—C22—H22A	109.6	C53—C54—H54B	109.4
C23—C22—H22B	109.6	H54A—C54—H54B	108
C21—C22—H22B	109.6	C54—C55—C56	111.42 (15)
H22A—C22—H22B	108.1	C54—C55—H55A	109.3
C24—C23—C22	111.40 (14)	C56—C55—H55A	109.3
C24—C23—H23A	109.3	C54—C55—H55B	109.3
C22—C23—H23A	109.3	C56—C55—H55B	109.3
C24—C23—H23B	109.3	H55A—C55—H55B	108
C22—C23—H23B	109.3	C55—C56—C51	110.01 (14)
H23A—C23—H23B	108	C55—C56—H56A	109.7
C25—C24—C23	112.04 (14)	C51—C56—H56A	109.7
C25—C24—H24A	109.2	C55—C56—H56B	109.7
C23—C24—H24A	109.2	C51—C56—H56B	109.7
C25—C24—H24B	109.2	H56A—C56—H56B	108.2
C23—C24—H24B	109.2	C66—C61—C62	111.98 (14)
H24A—C24—H24B	107.9	C66—C61—P2	110.77 (11)
C24—C25—C26	112.27 (16)	C62—C61—P2	114.60 (12)
C24—C25—H25A	109.2	C66—C61—H61	106.3
C26—C25—H25A	109.2	C62—C61—H61	106.3
C24—C25—H25B	109.2	P2—C61—H61	106.3
C26—C25—H25B	109.2	C63—C62—C61	112.06 (14)
H25A—C25—H25B	107.9	C63—C62—H62A	109.2
C21—C26—C25	109.64 (14)	C61—C62—H62A	109.2
C21—C26—H26A	109.7	C63—C62—H62B	109.2
C25—C26—H26A	109.7	C61—C62—H62B	109.2
C21—C26—H26B	109.7	H62A—C62—H62B	107.9
C25—C26—H26B	109.7	C62—C63—C64	112.95 (15)
H26A—C26—H26B	108.2	C62—C63—H63A	109
C36—C31—C32	110.94 (13)	C64—C63—H63A	109
C36—C31—P1	110.12 (11)	C62—C63—H63B	109
C32—C31—P1	113.99 (11)	C64—C63—H63B	109
C36—C31—H31	107.1	H63A—C63—H63B	107.8
C32—C31—H31	107.1	C65—C64—C63	110.73 (15)
P1—C31—H31	107.1	C65—C64—H64A	109.5
C33—C32—C31	111.49 (13)	C63—C64—H64A	109.5
C33—C32—H32A	109.3	C65—C64—H64B	109.5
C31—C32—H32A	109.3	C63—C64—H64B	109.5
C33—C32—H32B	109.3	H64A—C64—H64B	108.1
C31—C32—H32B	109.3	C64—C65—C66	111.66 (15)
H32A—C32—H32B	108	C64—C65—H65A	109.3
C34—C33—C32	111.37 (14)	C66—C65—H65A	109.3
C34—C33—H33A	109.4	C64—C65—H65B	109.3
C32—C33—H33A	109.4	C66—C65—H65B	109.3
C34—C33—H33B	109.4	H65A—C65—H65B	107.9
C32—C33—H33B	109.4	C65—C66—C61	111.43 (14)
H33A—C33—H33B	108	C65—C66—H66A	109.3
C35—C34—C33	111.08 (14)	C61—C66—H66A	109.3
C35—C34—H34A	109.4	C65—C66—H66B	109.3
C33—C34—H34A	109.4	C61—C66—H66B	109.3
C35—C34—H34B	109.4	H66A—C66—H66B	108
C33—C34—H34B	109.4	Cl3—C67—Cl2	110.91 (11)
H34A—C34—H34B	108	Cl3—C67—H67A	109.5
C34—C35—C36	111.60 (13)	Cl2—C67—H67A	109.5
C34—C35—H35A	109.3	Cl3—C67—H67B	109.5
C36—C35—H35A	109.3	Cl2—C67—H67B	109.5
C34—C35—H35B	109.3	H67A—C67—H67B	108
C36—C35—H35B	109.3	C11—P1—C21	105.27 (7)
H35A—C35—H35B	108	C11—P1—C31	104.38 (7)
C35—C36—C31	110.80 (13)	C21—P1—C31	106.28 (7)
C35—C36—H36A	109.5	C11—P1—Ag1	116.62 (5)
C31—C36—H36A	109.5	C21—P1—Ag1	112.35 (5)
C35—C36—H36B	109.5	C31—P1—Ag1	111.12 (5)
C31—C36—H36B	109.5	C41—P2—C51	105.46 (8)
H36A—C36—H36B	108.1	C41—P2—C61	104.51 (8)
C46—C41—C42	118.71 (15)	C51—P2—C61	107.21 (8)
C46—C41—P2	118.77 (13)	C41—P2—Ag1	116.45 (6)
C42—C41—P2	122.31 (13)	C51—P2—Ag1	110.23 (5)
C43—C42—C41	120.50 (16)	C61—P2—Ag1	112.34 (5)
C43—C42—H42	119.7	O3—Cl1—O1	109.49 (9)
C41—C42—H42	119.7	O3—Cl1—O2	109.47 (10)
C44—C43—C42	120.04 (16)	O1—Cl1—O2	109.74 (11)
C44—C43—H43	120	O3—Cl1—O2	109.47 (10)
C42—C43—H43	120	O1—Cl1—O2	109.74 (11)
C45—C44—C43	119.93 (16)	O3—Cl1—O4	109.73 (10)
C45—C44—H44	120	O1—Cl1—O4	111.51 (11)
C43—C44—H44	120	O2—Cl1—O4	106.87 (10)
C44—C45—C46	120.16 (16)	O2—Cl1—O4	106.87 (10)
C44—C45—H45	119.9	P2—Ag1—P1	177.473 (18)
			
C16—C11—C12—C13	0.9 (2)	C63—C64—C65—C66	55.8 (2)
P1—C11—C12—C13	−175.22 (13)	C64—C65—C66—C61	−56.3 (2)
C11—C12—C13—C14	−0.8 (3)	C62—C61—C66—C65	53.60 (19)
C12—C13—C14—C15	−0.5 (3)	P2—C61—C66—C65	−177.12 (12)
C13—C14—C15—C16	1.6 (3)	C12—C11—P1—C21	−153.34 (13)
C14—C15—C16—C11	−1.5 (3)	C16—C11—P1—C21	30.73 (15)
C12—C11—C16—C15	0.3 (2)	C12—C11—P1—C31	94.94 (14)
P1—C11—C16—C15	176.25 (13)	C16—C11—P1—C31	−80.99 (14)
C26—C21—C22—C23	59.98 (17)	C12—C11—P1—Ag1	−28.05 (15)
P1—C21—C22—C23	−176.65 (11)	C16—C11—P1—Ag1	156.02 (11)
C21—C22—C23—C24	−56.32 (18)	C26—C21—P1—C11	−165.15 (12)
C22—C23—C24—C25	52.8 (2)	C22—C21—P1—C11	72.80 (12)
C23—C24—C25—C26	−52.9 (2)	C26—C21—P1—C31	−54.78 (13)
C22—C21—C26—C25	−59.33 (19)	C22—C21—P1—C31	−176.83 (11)
P1—C21—C26—C25	178.38 (13)	C26—C21—P1—Ag1	66.94 (12)
C24—C25—C26—C21	56.3 (2)	C22—C21—P1—Ag1	−55.11 (12)
C36—C31—C32—C33	−54.80 (19)	C36—C31—P1—C11	−71.68 (12)
P1—C31—C32—C33	−179.80 (12)	C32—C31—P1—C11	53.75 (13)
C31—C32—C33—C34	54.67 (19)	C36—C31—P1—C21	177.33 (11)
C32—C33—C34—C35	−55.15 (19)	C32—C31—P1—C21	−57.24 (13)
C33—C34—C35—C36	56.25 (19)	C36—C31—P1—Ag1	54.82 (12)
C34—C35—C36—C31	−56.46 (18)	C32—C31—P1—Ag1	−179.74 (10)
C32—C31—C36—C35	55.37 (18)	C46—C41—P2—C51	−158.93 (14)
P1—C31—C36—C35	−177.48 (11)	C42—C41—P2—C51	26.34 (17)
C46—C41—C42—C43	3.1 (3)	C46—C41—P2—C61	88.20 (15)
P2—C41—C42—C43	177.85 (14)	C42—C41—P2—C61	−86.53 (16)
C41—C42—C43—C44	−5.1 (3)	C46—C41—P2—Ag1	−36.36 (16)
C42—C43—C44—C45	5.6 (3)	C42—C41—P2—Ag1	148.91 (13)
C43—C44—C45—C46	−4.3 (3)	C52—C51—P2—C41	64.00 (13)
C42—C41—C46—C45	−1.8 (3)	C56—C51—P2—C41	−173.39 (12)
P2—C41—C46—C45	−176.69 (14)	C52—C51—P2—C61	174.97 (11)
C44—C45—C46—C41	2.4 (3)	C56—C51—P2—C61	−62.42 (13)
C56—C51—C52—C53	57.40 (19)	C52—C51—P2—Ag1	−62.47 (12)
P2—C51—C52—C53	179.98 (13)	C56—C51—P2—Ag1	60.14 (12)
C51—C52—C53—C54	−56.3 (2)	C66—C61—P2—C41	−69.12 (13)
C52—C53—C54—C55	55.7 (2)	C62—C61—P2—C41	58.75 (14)
C53—C54—C55—C56	−56.1 (2)	C66—C61—P2—C51	179.26 (11)
C54—C55—C56—C51	56.4 (2)	C62—C61—P2—C51	−52.87 (14)
C52—C51—C56—C55	−57.00 (19)	C66—C61—P2—Ag1	58.02 (12)
P2—C51—C56—C55	−179.26 (13)	C62—C61—P2—Ag1	−174.11 (10)
C66—C61—C62—C63	−51.22 (19)	O2—O2—Cl1—O3	0.0 (2)
P2—C61—C62—C63	−178.47 (12)	O2—O2—Cl1—O1	0.0 (2)
C61—C62—C63—C64	51.6 (2)	O2—O2—Cl1—O4	0.00 (19)
C62—C63—C64—C65	−53.8 (2)		

### Hydrogen-bond geometry (Å, °)

**Table:**

*D*—H···*A*	*D*—H	H···*A*	*D*···*A*	*D*—H···*A*
C23—H23A···O3^i^	0.99	2.48	3.394 (2)	153
C67—H67A···O2	0.99	2.52	3.423 (3)	152
C13—H13···O3^ii^	0.95	2.54	3.448 (2)	160

Symmetry codes: (i) *x*−1/2, −*y*+1, *z*−1/2;  (ii) *x*−1, *y*, *z*.

## References

[bb1] Altomare, A., Burla, M. C., Camalli, M., Cascarano, G. L., Giacovazzo, C., Guagliardi, A., Moliterni, A. G. G., Polidori, G. & Spagna, R. (1999). *J. Appl. Cryst.* **32**, 115–119.

[bb2] Alyea, E. C., Ferguson, G. & Somogyvari, A. (1982). *Inorg. Chem.* **21**, 1369–1371.

[bb3] Alyea, E. C., Kannan, S. & Meehan, P. R. (2002). *Acta Cryst.* C**58**, m365–m367. 10.1107/s0108270102007734 12094028

[bb4] Baiada, A., Jardine, F. H. & Willett, R. D. (1990). *Inorg. Chem.* **29**, 3042–3046.

[bb5] Bayler, A., Schier, A., Bowmaker, G. A. & Schmidbaur, H. (1996). *J. Am. Chem. Soc.* **118**, 7006–7007.

[bb6] Berners-Price, S. J., Bowen, R. J., Harvey, P. J., Healy, P. C. & Koutsantonis, G. A. (1998). *J. Chem. Soc. Dalton Trans.* pp. 1743–1750.

[bb7] Bowmaker, G. A., Harvey, P. J., Healy, P. C., Skelton, B. W. & White, A. H. (1996). *J. Chem. Soc. Dalton Trans.* pp. 2449–2465.

[bb8] Brandenburg, K. & Putz, H. (2005). *DIAMOND* Crystal Impact GbR, Bonn, Germany. Bruker (2009).

[bb9] Bruker (2007). *APEX2*, *SAINT-Plus*, *XPREP* and *SADABS* Bruker AXS Inc., Madison, Wisconsin, USA.

[bb10] Burgoyne, A. R., Meijboom, R., Muller, A. & Omondi, B. (2010). *Acta Cryst.* E**66**, m503–m504. 10.1107/S1600536810011724 PMC2979226 21579003

[bb11] Camalli, M. & Caruso, F. (1988). *Inorg. Chim. Acta*, **144**, 205–211.

[bb12] Cingolani, A., Pellei, M., Pettinari, C., Santini, C., Skelton, B. W. & White, A. H. (2002). *Inorg. Chem.* **41**, 6633–6645. 10.1021/ic020375g 12470058

[bb13] Farrugia, L. J. (1997). *J. Appl. Cryst.* **30**, 565.

[bb14] Farrugia, L. J. (1999). *J. Appl. Cryst.* **32**, 837–838.

[bb15] Fenske, D., Rothenberger, A. & Wieber, S. (2007). *Eur. J. Inorg. Chem.* pp. 648–651.

[bb16] Flack, H. D. (1983). *Acta Cryst.* A**39**, 876–881.

[bb17] Liu, J. J., Galetis, P., Farr, A., Maharaj, L., Samarasinha, H., McGechan, A. C., Baguley, B. C., Bowen, R. J., Berners-Price, S. J. & McKeage, M. J. (2008). *J. Inorg. Biochem.* **102**, 303–310. 10.1016/j.jinorgbio.2007.09.003 18029019

[bb18] Meijboom, R., Bowen, R. J. & Berners-Price, S. J. (2009). *Coord. Chem. Rev.* **253**, 325–342.

[bb19] Muetterties, E. L. & Alegranti, C. W. (1972). *J. Am. Chem. Soc.* **94**, 6386–6391.

[bb20] Pauling, L. (1960). *The Nature of the Chemical Bond* 3rd ed., pp. 224–256. Ithaca: Cornell University Press.

[bb21] Sheldrick, G. M. (2008). *Acta Cryst.* A**64**, 112–122. 10.1107/S0108767307043930 18156677

